# Application of a Mixed Methods Multi-Criteria Decision Analysis Framework in Integrated Health Care

**DOI:** 10.5334/ijic.5997

**Published:** 2022-06-10

**Authors:** Robin Blythe, Hannah Carter, Bridget Abell, David Brain, Carly Dyer, Nicole White, Sanjeewa Kularatna, Steven McPhail

**Affiliations:** 1 Australian Centre for Health Services Innovation, Centre for Healthcare Transformation, School of Public Health and Social Work, Faculty of Health, Queensland University of Technology, Australia

**Keywords:** integrated care, health economics, multi-criteria decision analysis, economic evaluation

## Abstract

**Background:**

Evaluating integrated care programs is complex. Integration benefits may not become apparent within short evaluation timeframes, and many programs provide a wide variety of health and non-health benefits. To address these challenges, we illustrate a mixed methods approach for evaluating multiple integrated care programs using multi-criteria decision analysis.

**Methods:**

We adapted a decision support tool used by local decision makers to compare data extracted from 17 different integrated care evaluations. Criteria included impact on health services capacity, patient outcomes, integration of care, workforce development and implementation risk, weighted based on stakeholder preferences. Program benefits were compared to their implementation costs, and assessed using cost-effectiveness methods. Sensitivity analysis examined the impact of different criteria weights.

**Results and discussion:**

This method captured a diverse range of benefits provided by integrated care programs and provided an accessible heuristic to compare many projects simultaneously. However, this approach may not be sensitive to the appropriateness of each criterion to the health system, the magnitude of difference in individual criteria, equity considerations or socio-political factors. Internal and external validation, especially for subjective criteria such as implementation risk, are needed.

**Conclusions:**

This work offers a feasible, flexible and pragmatic approach for evaluating integrated care programs.

## Background

Integrated care to overcome health system fragmentation and provide attentive patient-centred healthcare is an important objective in contemporary healthcare delivery [1]. It has been consistently identified as a priority for health services reform to ensure health systems can continue to meet patient needs while managing rising costs [2,3]. Integrated care has also been recognised as an important mechanism by which to achieve the *quadruple aim* of health system optimisation: enhancing patient experience, improving population health, reducing costs, and improving the work life of health care providers [4]. The World Health Organization (WHO) defines integrated care as “the organisation and management of health services so that people get the care they need, when they need it, in ways that are user-friendly, achieve the desired results and provide value for money” [5]. It further describes integration as the delivery of the “continuum of health promotion, diagnosis, treatment, disease management, rehabilitation and palliative care services, at the different levels and sites of care within the health system, and according to their needs, throughout their whole life” [6]. Common concepts in integrated care frameworks cite additional values such as person-centredness, sustainability and transparency [7], suggesting that a broad umbrella of terms and ideas can constitute integration [8].

The practical implementation and evaluation of integrated care is challenging due to its conceptual ambiguity and complex interplay of systems and individuals. The WHO definitions above encompass more information than is typically feasible to evaluate in the scope of assessing health services. Also, long term improvements in health outcomes and efficiency gains which may occur years later are often not visible in the short turnarounds typically required for health services evaluation to align with funding cycles [9]. While some indicators do exist to measure individual domains of integrated care [10], quantifying and combining these domains to assess overall integration of programs is challenging. Additionally, the wide variety of outcomes resulting from integration may not be readily compared across projects or settings. For example, a scoping review identified both the Greek Open Care Centres for the Elderly program, which provided older adults with comprehensive primary care in the home, and the New Zealand Healthy Housing Programme to reduce housing-related health issues such as poor ventilation, as examples of integrated care initiatives [11]. However, if both were competing for the same limited health budget, summarising and comparing the diverse benefits would be challenging. The complexity of integrated care requires evaluation of not only effectiveness and cost-effectiveness, but also an understanding of the mechanisms of action: what worked, what did not, and why, during implementation [8]. These concepts can also help to understand what constitutes integration in the applied setting.

This paper presents an approach to address these issues by synthesising quantitative and qualitative information in a flexible multi-criteria decision analysis (MCDA) framework to examine and evaluate integration in an applied healthcare setting. In this context, MCDA is defined as the evaluation of multiple independent outcomes, weighted by decision makers’ preferences and aggregated into a single estimate of total benefit. The use of MCDA in health services research is increasing. Health technology assessments, economic evaluations and priority setting have all been reviewed using this technique in situations where a simple cost-effectiveness or cost-benefit calculation is deemed too narrow in scope [12,13,14,15]. This is a potentially suitable framework for assessing integration, where benefits may be intangible in the short term and when longer evaluation periods are impractical.

The aim of this study was to describe this MCDA framework and examine its application and suitability for evaluating 17 health services projects, which were funded to test new models of integrated care.

## Methods

### Setting

In 2016, the Minister for Health in Queensland, Australia, announced an investment of $35 million AUD to support integrated models of healthcare. The Integrated Care Innovation Fund (ICIF) was designed to support publicly funded Hospital and Health Services to work in partnership with their local Primary Health Networks (PHNs), government departments, not-for-profit groups and private sector providers to develop and test novel approaches to integrated care. The ICIF used a region-specific approach in which health services designed interventions for their local populations, with the goal of assessing feasibility and sustainability of future state-wide implementation. Twenty-three health innovation projects were funded, spanning 13 of the 16 Hospital and Health Services (HHSs) and six of the seven PHNs across Queensland. Projects were selected by the state government using the following criteria: relevance to large populations in Queensland, enhancement of patient experiences, potential cost-savings, and expected improvement in experience and workload of healthcare providers. Of these 23 projects, 17 delivered evaluable models of care. Projects are summarised in Table 1, classified according to geographic remoteness [16] and level of integration [17]. Integration level is described in Table 3. Projects addressed a wide variety of populations and chronic health conditions including Hepatitis C, frailty, and mental health.

**Table 1:** List of integrated care projects by location, key intervention components, and level of integration from the conceptual framework for integrated care.


PROJECT NUMBER	PROJECT DESCRIPTION	LOCATION	KEY COMPONENT(S)	LEVEL OF INTEGRATION

1	Online pathway for the diagnosis, referral, and management of primary mental health care	Remote to very remote	Introduction and training for a stepped care mental health model in emergency departments Software platform to give providers shared access to patient information	Professional Organisational

2	Improving access and care planning for the management of COPD*	Major city	Creation of a multidisciplinary pulmonary rehabilitation pathway GP^†^ education and best practice adherence auditing for rehabilitation pathway components	Professional

3	Community outreach service for Hepatitis C virus diagnosis and treatment	Inner regional	Hub-and-spoke model in which a multidisciplinary telehealth team (hub) supported GPs and community workers to deliver care in the community, and nurses to lead community assessment and mobile liver imaging services (spokes)	Clinical Professional Organisational

4	Primary and secondary co-management of paediatric ADHD** patients	Major city	Weekly remote consultations between GPs and specialists to improve clinical confidence in managing ADHD patients within primary care	Professional

5	Integration of funding models for allied health in rural communities	Outer regional	Service coordination for allied health based on community needs Integration of funding streams Increased telehealth and allied health assistant access	Professional Organisational

6	Telehealth and emergency department redesign for partnerships between aged care facilities and emergency care	Outer regional	Dedicated emergency department team for low acuity presentations Telehealth assessment of aged care facility patients between emergency and aged care nurses to avoid unnecessary emergency presentations Secure patient data sharing service between hospitals and aged care facilities	Clinical Professional Organisational

7	Multidisciplinary clinics to treat patients with concomitant gastroenterological and hepatological symptoms	Major city	Identification and enrolment of applicable patients for 12-week care management pathway Multi-disciplinary, GP-led community monitoring of patients post-pathway	Clinical Professional

8	Teledentistry model for remote monitoring of dental caries using intraoral cameras	Very remote	Provision of intraoral cameras and data sharing service to enable on-site community workers and remote dentists to conduct telehealth assessment and referral	Professional Organisational

9	Multidisciplinary support teams for chronic respiratory diseases including allied health, home visiting services and patient education	Major city to inner regional	Specialist care hotline for GPs to consult with clinics for rapid referral Multidisciplinary care team and increased allied health support to provide home visits and education	Clinical Professional

10	Novel linkages between acute and community-based services for cognitively impaired older persons	Outer regional	Emergency department screening to identify and redirect elderly to more appropriate services Specialist outreach for community-dwelling elderly	Clinical Professional

11	Older persons enablement and rehabilitation for complex health conditions	Outer regional	Integration of primary and secondary care to create a shared management structure for complex older patients Early intervention and outreach service for patients at risk of imminent deterioration and hospitalisation	Clinical Professional Organisational

12	Facilitating social work liaisons for cognitively impaired patients with complex guardianship status requiring tribunal	Major city	Appointment of one hospital-based and one tribunal-based coordinator to coordinate patient hearings Engagement with patients and guardians on tribunal process	Professional Organisational

13	Paediatric shared care model for children with developmental, behavioural, and learning difficulties	Inner regional	Centralised intake model for paediatric referrals Development and delivery of a GP Diploma of Child Health	Clinical

14	Delivering GP education and tools to manage health and developmental needs of children in out of home (foster) care	Major city	Data sharing platform for children’s health providers Health system navigators for children in out-of-home care Development and training for GP digital assessment tools to establish best practice and understand care needs of children in out-of-home care	Professional Organisational

15	Integrating emergency, acute, and primary services for a patient-centred model of diabetes care	Inner regional	Aboriginal & Torres Strait Islander focused virtual team to plan post-referral care pathways Redirection of low acuity diabetes care to GPs, supported by additional primary care diabetes education and training Ambulance visits linked with diabetes educator to reduce unnecessary ambulance transfers	Clinical Professional Organisational

16	“One-stop-shop” model for the localisation and coordination of mental healthcare and social services	Inner regional	Centralised referral, triage, and treatment pathway for adults with mental illness Co-location of varied clinical and non-clinical services to enable patients to access requisite successively Shared provider/social work access to patient records to manage care and assess outcomes	Clinical Professional

17	Integrated diagnosis, management and discharge of frail elderly patients in hospital	Major city	Identification of admitted elderly at risk of functional decline to a multidisciplinary care ward Development of a comprehensive discharge plan engaging patient’s family and external care providers	Professional Organisational


* Chronic Obstructive Pulmonary Disease; ** Attention Deficit Hyperactivity Disorder; ^†^ General Practitioner.

### Evaluation and selected criteria

We evaluated all 17 projects individually over two years as contracted independent evaluators. In addition to this contracted work, we extracted relevant information from these evaluations to populate our MCDA tool. No additional data were collected for this paper that were not already extracted from each completed evaluation. Projects were evaluated prospectively, with a health economist and implementation scientist assigned to each. All data collection tools were specified at baseline, distributed when possible both pre- and post-implementation by clinical and administrative partners to patients and providers. Utilisation data to estimate capacity savings was collected from HHS partners where required, typically from the Queensland Hospital Admitted Patient Data Collection system. Net costs were used to determine the relative value generated by each domain. Implementation costs were collected by tallying all labour, equipment and location rental expenditure, including market rate valuation when contributions were given in-kind. Interview and focus group data from clinical and administrative partners was collected by qualitative experts retrospectively, with an emphasis on perceptions of integration and the barriers and facilitators to implementation.

We based our evaluation criteria on a previously developed MCDA framework, [12] modified to better align with the concept and objectives of integrated care. Based on the Quadruple Aim of health care optimisation [18] and the WHO global strategy on integrated care, [6,19] five criteria were selected by the authors and ICIF stakeholders. These criteria included improving health services capacity through shifting to lower acuity care; improving patient outcomes, including care accessibility, satisfaction, and health-related quality of life; integration of care to improve coordination, collaboration, and co-production; workforce development to improve the working life of care providers; and organisational risk, to ensure that care was sustainable in the long term. The criteria and associated outcome measures are outlined in Table 2.

**Table 2:** The five health services evaluation criteria across ICIF projects.


CRITERIA	OUTCOME MEASURES

Health service capacity	Services appropriately redirected from acute or emergency to primary or outpatient

	Length of stay in hospital or emergency department

Patient outcomes	Patient satisfaction

	Health-related quality of life

	Healthcare accessibility

Integration of care	Clinical: Evidence of greater patient-centred care, including patient engagement and care coordination

	Professional: Evidence of increased intra-professional partnerships, and shared care between providers

	Organisational: Evidence of greater cohesion in continuum of care and improved coordination across care organisations and networks

Workforce development	Provider satisfaction with workload, support, and quality of care

	Provider skills development for improved care delivery

Organisational risk	Implementation success relative to barriers and facilitators


These criteria were relevant to local health service decision makers and were able to be evaluated within a two-year period of project implementation. We determined that while integration had many intangible benefits, the selected outcome measures could be feasibly quantified to support decision making in organisations pursuing value-based, patient-centred care.

The framework allowed for criteria to be weighted to reflect their relative importance to decision makers. The Queensland Health department steering committee members tasked with overseeing the ICIF, including executives, administrators and patient advocates were asked to rate each criterion to inform the relative weightings applied across the criteria. Committee members chose to weight all objectives equally for this evaluation. For each criterion, an outcome was assigned for each project based on the independent evaluation. Each outcome was transformed with linear scaling to return a score between zero and two. Outcomes were assessed for duplication. For example, shorter length of stay could be associated with both healthcare capacity, as beds are available earlier, and patient outcomes, as typically healthier patients are discharged. To avoid scoring projects twice for the same outcome, the most immediate outcome, in this case healthcare capacity, determined which criteria it addressed. Net health services costs were then divided by the combined criteria scores to determine relative value for money.

We made several assumptions, supported by evidence, about how these criteria informed the effectiveness of integration in an applied health services context. First, we assumed that integration could reduce the frequency and duration of acute care through improvements in the coordination of different care providers [20,21]. In other words, we assumed that shifting care from ambulatory and acute settings to primary settings was an expected and desirable outcome, increasing health service capacity. Second, that any changes to service delivery should account for both clinical and patient outcomes, and that patient self-reporting via questionnaires on health-related quality of life and satisfaction were the best source for whether these changes were meaningful to patients [22,23]. We also assumed that a well-supported healthcare workforce was required to determine whether integration was able to improve provider skills and workloads, and that providers were best placed to decide whether these changes (workforce development) were amenable [24,25].

### Health service capacity

We assessed changes to health service capacity using two outcome measures: whether services were appropriately redirected from acute or emergency care to primary or ambulatory care, and whether there was a reduction in acute or emergency length of stay. If evaluation found a statistically significant reduction in the quantity of acute services rendered to patients, the project scored a two. If a reduction was observed but it was not statistically significant, the project scored a one. If there were no capacity savings, or capacity increased in one area but fell in another without proof of net positive project impact, the project scored a zero. Our definition for statistical significance was a p-value of less than 0.05.

### Patient outcomes

Patient outcomes were quantified using three measures: patient satisfaction, health related quality of life (QoL), and healthcare access. The Patient Satisfaction Questionnaire Short-form (PSQ-18) [26] and the EQ-5D were encouraged for use, though some projects preferred to apply more case-specific tools such as the St. George Respiratory Questionnaire [27]. A statistically significant improvement in patient satisfaction and/or QoL each earned a score of two for patient satisfaction and QoL, respectively. An observed but not statistically significant improvement earned a score of one, and no changes or unmeasured outcomes earned a score of zero. Access was deemed binary due to challenges in quantifying accessibility, with zero indicating no improvement and one indicating a perceived improvement by patients. Projects could score up to five and were multiplied by 2/5 to be consistent with other outcomes.

### Integration of care

We assessed three of six recognised domains of integrated care which were most relevant to the ICIF program goals: clinical, professional and organisational integration [17]. However, measuring the degree of integration was challenging due to its conceptual ambiguity. We were unable to find a measurement system that quantitatively assessed whether integration had occurred, so we used a combination of quantitative and qualitative data to determine an outcome. Two evaluators assessed health service outcomes and implementation data from each project, seeking evidence of integration across each domain. If evidence was agreed by both evaluators, the project received two points for each domain in which integration was observed, for a maximum of six points. Projects received a score of zero where no evidence of integrated care was observed, or data was not available for scoring. Scores were then divided by three to be consistent with other outcomes. The integration domains are explained further in Table 3.

**Table 3:** Definitions and examples of integration used in evaluating each project.


INTEGRATION DOMAIN	DEFINITION [17]	IMPLEMENTATION IN PRACTICE	EXAMPLES FROM PROJECTS

Clinical	Coherence in the primary process of care delivery to individual patients	Care is designed around the needs of the patient and addresses a range of factors contributing to patient health. Users are actively engaged as partners to improve their own well-being.	– Providing mobile services and triage to patients with mobility restrictions – Creating a single point of care for patients with complex care needs – Co-locating social services with mental health care delivery

Professional	Partnerships between professionals both within and between healthcare organisations	Care involves a range of providers, across multiple specialities, modalities, or locations with a shared vision to improve healthcare delivery.	– Facilitating specialist telehealth consults to improveprimary care provision – Creating multidisciplinary shared care plans for mentalhealth patients – Collaboratively developing elderly patient discharge plans with agedcare facilities

Organisational	Collective action across the entire care continuum	Interorganisational relationships, knowledge sharing, alliances, contracting and common mechanisms for governance and evaluation are observed, not necessarily limited to healthcare.	– Extending existing networks, such as with the local correctional centre, a key site for implementation – Breaking down silos that existed between the hospital- and community-based diabetes nursing services – Open communication about the scope of practice and needs of various service organisations


### Workforce development

We identified two workforce development outcomes that exemplified the fourth tenet of the quadruple aim of healthcare optimisation [18]. These were: (a) workforce sustainability, or how providers perceived the burden and fulfilment of their roles, and (b) quality of care provision, or the depth and breadth with which care was delivered [28,29]. Projects that both upskilled providers and improved the self-satisfaction with which they delivered care scored two. Projects that did one of these, but not both, scored one. Projects that did not upskill providers, or did so at the expense of increased workload or reduced self-satisfaction, scored zero.

### Organisational risk

The contextual conditions relative to implementation success are important to consider for the acceptability and sustainability of projects once the implementation phase has ended, and thus relate to the potential risks of funding each project. The ease of implementation in terms of facilitators and barriers, and their impact on success and sustainability, was a key evaluation component in ICIF project evaluation. For example, successful projects in challenging environments were often implemented through work-arounds or top-down approaches that were difficult to sustain beyond the attentions of their advocates. We developed a standard risk matrix that prioritised projects which demonstrated successful implementation in the context of a welcoming environment.

Assessment of environmental barriers and facilitators, and perceptions of implementation success were conducted through qualitative evaluation. This evaluation was guided by the Consolidated Framework for Implementation Research (CFIR) [30], a widely cited and rigorously developed determinants framework for implementation, which applied a categorisation structure across the qualitative data. Data were captured through implementation diaries/logs, surveys, semi-structured interviews and focus groups with project stakeholders and implementation teams. In total, 134 stakeholders provided these data across the 17 projects evaluated.

**Figure 1:**
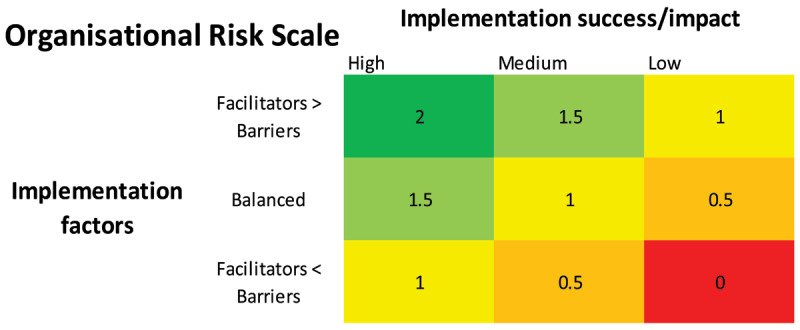
Organisational risk scale of project implementation.

To enable a valuation of implementation risk and environment for each project we tabulated the number of facilitators and barriers across all CFIR domains for each project. If facilitators outweighed barriers by more than 25%, the project scored in the top row of the Organisational Risk Scale matrix in Figure 1. If CFIR barriers outweighed facilitators by more than 25%, the project scored in the bottom row. All other projects were considered balanced and scored in the middle row.

To assess implementation success, we examined qualitative interview data, project logs and plans, sought project evaluator opinion, and reviewed implementation outcomes. If these data suggested that the project had achieved more than 2/3 of its implementation objectives, success was deemed high and it scored in the first column of Figure 1. Projects achieving less than 1/3 of their objectives scored low on impact. Ratios in between scored in the middle row. Scores ranged from zero to two in 0.5 increments in which higher scores corresponded to greater likelihood of long-term changes to the model of care. The risk scale is shown in Figure 1.

### Costs

Implementation costs were taken from project budgets, less any amounts retained by the health service at the close of the financial year. In-kind contributions, or goods and services delivered free of charge or heavily discounted, were valued at the market rate and added to the gross cost of service delivery. To avoid double-counting, only cost-savings that could not be explained by capacity improvements, such as from averted hospital admissions, were recorded.

### Sensitivity analysis

To assess the degree to which criteria weighting could affect final scores, we created three alternative sets of ratings to assess the MCDA evaluation framework. We ranked the criteria through three lenses: 1) quantitative focus, 2) qualitative focus, and 3) a rating based on the authors’ perceptions of steering committee priorities. The purpose of this sensitivity analysis was not to attach meaningful value to any particular rating scheme, but to determine the range of possible values for projects depending on their merits and the preferences of decision makers.

For quantitative focused ratings, we rated capacity, patient outcomes, workforce development, integration of care, and implementation risk as one through five, respectively, based on the relative level of quantitative data to support each criterion. For qualitative focused ratings, we reversed the order. For the final rating, we rated capacity and outcomes joint first, followed by implementation risk, workforce outcomes and integration. We then compared each project’s cost-per-point across all four sets of alternate ratings and examined the range of returned values. The ranking methodology has been published elsewhere [12]. Criteria scores were multiplied by relative weights under different criteria rankings, then ordered by increasing cost-per-point to determine value.

## Results

### Health service capacity

Six projects demonstrated a statistically significant improvement in healthcare capacity. These were project 2 (18% reduction in admitted LOS, 2% reduction in hospitalisations per patient), project 6 (8% reduction in ED presentation rate), project 12 (35% reduction in admitted LOS), project 15 (26% reduction in admission rate from ED), project 16 (3% reduction in ED presentation rate, 14% reduction in admission rate from ED, 7% reduction in admitted LOS) and project 17 (48% reduction in admitted LOS). A further four projects noted improvements in capacity but had insufficient evidence to declare statistical significance, including projects 9 (6% reduction in readmission rate) and 10 (3% reduction in ED presentation rate). Results for one project did not contain the information needed to assess statistical significance. One project achieved a reduction in LOS but was associated with an increase in ED utilisation, indicating that the project may have shifted rather than reduced acute service use. The remaining seven projects did not successfully impact care capacity based on our defined criteria.

### Patient outcomes

Four projects found improved patient outcomes through a validated quality of life survey tool, including the EQ-5D-5L [31], SF-12 [32], or AQoL-8D [33], and used either means testing or regression to determine a highly likely improvement in quality of life. One project used Monte Carlo estimation with health utility from the literature to validate this change. In project 2, quality of life improvements were measured using the St George Respiratory Questionnaire [34], but this change was not considered statistically significant.

Data required to analyse before and after changes in patient satisfaction were only collected in project 15. The remaining projects were unable to measure patient satisfaction under the old model of care. Access improved for five projects (2, 8, 11, 15, 16) by reducing patient transportation times from their homes to different services, for four projects (5, 9, 12, 13) by reducing wait times, and for one project by allowing patients in the prison system to receive care.

### Integration of care

Clinical integration was successful across nine of 17 (53%) projects, with the remainder not demonstrating evidence of clinical integration for patient-centred care. Professional integration was successful for 14 of 17 (82%) projects, with a further three projects achieving partial professional integration. Organisational integration was successful for eight of 17 (47%) projects, with one additional project achieving partial integration. Less than a quarter of projects (4/17) achieved integration across all three domains; however, all projects achieved partial integration in at least one domain.

### Workforce development

Workforce development was observed in eleven of 17 (65%) projects. Of these, ten included either staff training or an increased focus on delivering better quality care, which was supported by provider opinions. No projects were classified as leading to an overall decline in job satisfaction. Six projects reported improving the job satisfaction of the providers involved. Increased workloads accompanying the interventions were reported to have prevented more significant improvements in overall job satisfaction.

### Organisational risk

**Figure 2:**
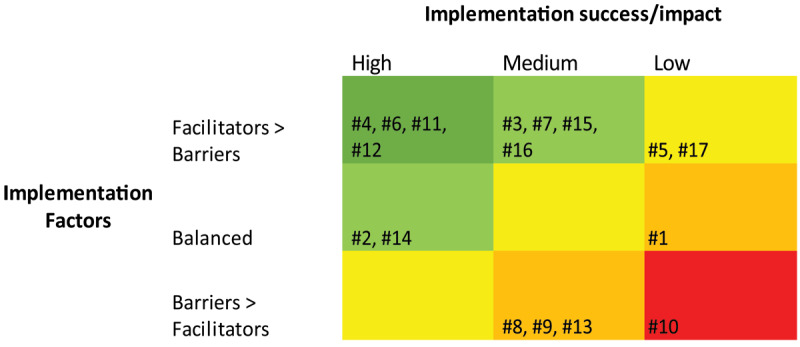
Organisational risk matrix of implementation environment vs implementation success for all projects.

Organisational risk, or threats to implementation and long-term sustainability, were low and implementation success high for four projects (4, 6, 11 and 12), scoring the full two points. Only one project was determined to have had a hostile implementation environment and to have failed implementation, scoring zero (project 10). The remaining projects scored between 0.5 to 1.5 depending on how successful implementation was in the face of barriers to success (Figure 2).

### MCDA Matrix

Each of the five criteria are shown in Table 4, with an indicator of value for money from dividing the net costs of the project by the total points.

**Table 4:** MCDA with equal weighting, sorted by cost per point. Fractions are rounded to the nearest decimal point.


PROJECT	CAPACITY	OUTCOMES	INTEGRATION	WORKFORCE	RISK	TOTAL	NET COST	COST PER POINT

4	0	0	0.7	1	2	3.7	$210,950	$57,014

5	0	0.4	1.3	0	1	2.7	$238,476	$88,324

15	2	1.2	2.0	2	1.5	8.7	$784,865	$90,214

14	0	0	1.3	2	1.5	4.8	$471,029	$98,131

11	1	1.2	2.0	1	2	7.2	$913,336	$126,852

17	2	1.2	1.3	1	0.5	6.0	$821,383	$136,897

3	1	1.2	2.0	0	1.5	5.7	$850,006	$149,124

12	2	0.4	1.3	0	2	5.7	$889,698	$156,087

10	0	0	1.0	0	0	1.0	$162,954	$162,954

7	0	0.8	1.3	2	1.5	5.6	$1,362,603	$243,322

9	1	0.4	1.3	0	0.5	3.2	$792,507	$247,658

2	2	1.2	0.7	2	1.5	7.4	$1,842,953	$249,048

13	0	0.8	0.7	1	0.5	3.0	$786,052	$262,017

6	2	0.4	2.0	1	2	7.4	$2,048,999	$276,892

16	2	1.6	1.3	2	1.5	8.4	$2,411,938	$287,135

8	0	0.4	1.0	1	0.5	2.9	$1,277,109	$440,382

1	0	0	0.7	0	0.5	1.2	$1,675,243	$1,396,036


### Cost-effectiveness

**Figure 3:**
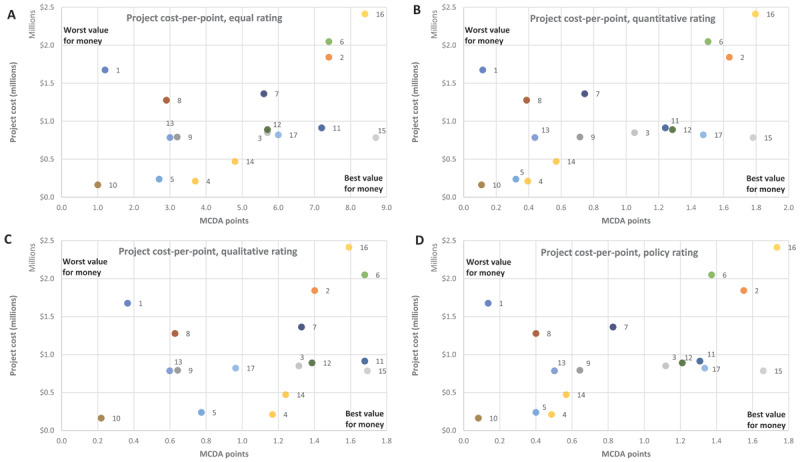
MCDA cost-per-point presented on a cost-effectiveness plane for panels **(A)** equal rating, **(B)** quantitatively oriented rating, **(C)** qualitatively oriented rating, and **(D)** policy analyst suggested rating.

The MCDA scores and cost pairs were plotted on a cost-effectiveness plane to allow for visualisation of these results (Figure 3). The horizontal axis is the MCDA score associated with each project, with higher scoring projects appearing towards the right-hand side of the figure. The vertical axis is each project’s overall cost, with higher cost projects appearing towards the top of the figure. Projects achieving the best outcomes for the lowest costs, thus representing the best value for decision makers, will be those closest to the bottom-right of the graph.

Projects were ranked by increasing cost-per-point (Table 4). The projects on the cost-effectiveness frontier, or the ones that provided the best value for money, were projects 10, 4, 14 and 15. No projects were both cheaper and higher scoring than usual care. No projects were cost saving, or below the X-axis.

### Alternative weightings

Under alternative weighting allocations, there was a marked variability in scores for several projects. Projects that were expensive, such as 6, or highly concentrated in one or two fields, such as 10, were significantly affected by alternate weightings. An expensive project was considered reasonable when it delivered strongly on a prioritised metric, but a cheaper project that delivered on less prioritised outcomes, such as project 5 and its score under quantitatively-oriented weighting, was penalised. In contrast, projects that were disproportionately expensive or weak in all fields, such as project 1 or 8, were not salvaged by different rating systems. Table 5 demonstrates how project ranking was affected under different ratings, with a rank of 1 providing the best value and 17 the worst.

**Table 5:** List of four alternative weighting paradigms and observed range (minimum/maximum).


PROJECT	RANK (COST PER POINT)				RANGE
	
	UNWEIGHTED	QUANTITATIVE	QUALITATIVE	AUTHOR PERCEPTIONS	MINIMUM, MAXIMUM

1	17	17	17	17	[17,17]

2	12	10	14	9	[9,14]

3	7	7	7	7	[7,7]

4	1	2	1	1	[1,2]

5	2	6	2	3	[2,6]

6	14	4	11	12	[4,14]

7	10	15	10	14	[10,15]

8	16	16	16	16	[16,16]

9	11	9	12	10	[9,12]

10	9	13	8	15	[8,15]

11	5	5	5	5	[5,5]

12	8	4	6	6	[4,8]

13	13	14	13	13	[13,14]

14	4	8	3	8	[3,8]

15	3	1	4	2	[1,4]

16	15	11	15	11	[11,15]

17	6	3	9	4	[3,9]


## Discussion

This study has demonstrated the potential for an MCDA framework to be applied when performing a holistic evaluation of different integrated care initiatives. This approach provides a framework for summarising and comparing implementation and healthcare outcomes through a mixed-methods approach, with a potential to apply preference-based stakeholder weightings to guide policy decisions.

Health service capacity, patient outcomes and workforce development were straightforward to rate using the MCDA framework, requiring little time for two reviewers to extract data from the individual project evaluations that had been completed. However, integration of care and organisational risk were more challenging, requiring substantial time and effort as the reviewers were required to extract additional qualitative data from transcripts. This process was partly enabled by having the same staff on both the original evaluations and the MCDA framework. Original evaluations and the MCDA framework were all informed by the CFIR, which expedited the review process and showed the value of using a consistent implementation framework when evaluating multiple projects.

Few prior studies have recognised the potential benefits of using a MCDA approach to guide evaluation and decision making in integrated care, with most restricted to health technology assessment, priority setting, and funder decision-making [10,12,13,35]. Integrated care has been proposed as a means of achieving the Quadruple Aim of health care; its evaluation requires a broader set of criteria that are able to capture a wider range of health and non-health benefits. This includes changes to the systems and models of health care delivery as well as important patient, provider and health service outcomes. Accounting for these intermediate outcomes is important when evaluating integrated care projects, due to the length of time it may take for interventions to demonstrate clinical benefit and the need for evidence to support decision making pertaining to recurrent funding. This has been demonstrated in a previous MCDA framework developed to evaluate a suite of integrated care projects for people with multi-morbidity in Europe [36]. The scope of that framework was adapted to include clinical benefit, cost, patient reported outcome and experience measures, as well as selected health service outcomes. However, there has been limited assessment of the fourth aim, improved clinician experience, into any previously reported MCDA frameworks. There has also been no prior attempt to include implementation or process outcomes in a MCDA framework for integrated care evaluation. These outcomes are particularly important to capture in the context of integrated care initiatives which often involve system-level changes to health service delivery, impacting multiple stakeholders.

To address these gaps in the literature, the set of evaluation criteria from the present study addressed each of the Quadruple Aims that collectively encompassed patient, provider and health service perspectives. The criteria reflect the impact of integrated care initiatives in improving the quality of health care delivery and achieving recognised health service priorities. The funder was not able to determine the degree of integration from the funding proposals submitted by different HHSs, so we created the integration of care criteria to adequately capture this concept across each program.

We chose to explicitly incorporate two other unique criteria into the MCDA which we considered to be important when evaluating integrated care projects: success in achieving integration of care, and organisational implementation risk. Integration has been linked to downstream positive outcomes that were outside the scope of the evaluation window [37,38]. This was particularly important as qualitative and quantitative evaluation of projects demonstrated considerable variability in achieving integration across all domains. Our inclusion of qualitative and quantitative information about implementation context, barriers, facilitators, and success as an evaluation criterion for decision making is also novel. While the adapted Evidence and Value: Impact on DEcisionMaking (EVIDEM) MCDA framework [39] includes a qualitative assessment of some contextual criteria including impact on health service capacity, fit with system, and political/cultural context, these do not act as standalone criteria but rather act to transform the value of interventions or projects within the MCDA.

A key component of evaluation is assessment of the processes and contextual factors that can support or impede the implementation, scale and sustainability of integrated care projects, [40]. This information offers important insights for decision makers about the likely sustainability, scalability and transferability of projects that cannot be gathered by the assessment of patient and health service outcomes alone. For example, while Project 10 was the most cost-effective in our evaluation, attempting to sustain or scale this intervention should be approached with caution due to its low implementation success and high proportion of contextual barriers. Conversely, Project 15 was both effective and easy to implement, indicating that it would likely be a good candidate for ongoing funding and expansion to other health services.

### Limitations

There are several limitations with the MCDA framework outlined in this study. The scoring system’s discrete nature does not distinguish between projects with small and large validated changes to outcomes, the number of patients affected, and the equity of these outcomes. The use of p-values as the measure of statistical validation may be considered quite simplistic and reductionist. We selected a conceptual approach to criteria selection and scoring because the breadth and scope of different projects often called for different types of data to be collected, and different measures of effectiveness and success. While this limited the ability of the MCDA in this study to compare outcomes on a relative basis, it was a necessary simplification and has been adopted by other MCDA tools [36]. For example, project 17, which reduced hospital length of stay in cognitively impaired patients by over three weeks, was scored comparably to project 6, which reduced ED presentation rate by 8%. Project 5, which was associated with a slight reduction in wait times through increased allied health availability, was scored comparably to project 3, which brought hepatitis C screening to patients who had never engaged with the health system before. Both equity and effect sizes were difficult to objectively quantify within a MCDA framework encompassing multiple dimensions and perspectives.

The use of qualitative information in the MCDA process is a novel component of this framework but could also lead to a lack of internal and external validity. While triangulated from several sources and with adequate sample sizes, assessment of successful integration and implementation risk were nevertheless subjective, and it is possible that values assigned may change between different groups of evaluators. However, in the absence of a recognised method for synthesising this information into a MCDA tool, we propose this as an interim method to include this important and often overlooked data.

The weighting system was considered politically fraught in a transparent governing system. Stakeholders expressed reluctance to being on record as prioritising any criteria over another. Despite explicitly presenting integration as a primary motivation of the ICIF, stakeholders declined to attach additional importance to integration as an outcome in and of itself. We attempted to address this by suggesting anonymity in stakeholder feedback, but this was declined by the executive group. A benefit of equal weighting was that the results were easy to interpret.

Future research using this MCDA framework, including localised adaptations of the approach described in this study, should focus on validation. Both internal and external validation are required to determine the tool’s suitability for measuring target criteria and applicability to other health systems, respectively. Additional research on the usefulness of the scores, weighting system and cost-effectiveness plane would also be beneficial in the context of a decision support framework.

### Strengths

This MCDA method demonstrates a transparent and flexible approach to evaluating disparate integrated care programs, allowing healthcare interventions with a variety of impacts to be compared on the same scale. It measures both quantitative and qualitative outcomes and provides a method of transformation to assess their relative merits when more specific approaches, including meta-analyses, are not possible. It also addresses impacts that have no direct measurable outcomes on patients or providers in the short term but are associated with higher quality care over the long term.

An advantage of this MCDA approach is that assumptions are explicitly defined in the scoring and rating systems. This allows for substantially broader application, as the methods can be challenged by a variety of stakeholders if unsuited to the context. In contexts such as health system decision support, community healthcare provision or short-term policy, it can provide intermediate findings and evidence prior to the long-term evaluation of novel programs. This MCDA may also be used as a supplement to standard evaluation processes, particularly if funding bodies want to account for benefits outside clinical effectiveness.

## Conclusions

The mixed methods multi-criteria decision analysis approach outlined in this study has potential for adoption as a holistic evaluation framework for integrated care programs. Both quantitative and qualitative measures were included with consideration of impacts that may not fall within feasible evaluation timeframes or policy windows. This MCDA framework was successfully applied in the evaluation of 17 wide-ranging integrated care initiatives in the state of Queensland, Australia. We propose that this MCDA has potential to be used as an intermediate evaluation framework prior to the long-term evaluation of integrated care initiatives.
