# Correction: Application of a Mixed Methods Multi-Criteria Decision Analysis Framework in Integrated Health Care

**DOI:** 10.5334/ijic.6980

**Published:** 2022-07-08

**Authors:** Robin Blythe, Hannah Carter, Bridget Abell, Megan Campbell, David Brain, Carly Dyer, Nicole White, Sanjeewa Kularatna, Steven McPhail

**Affiliations:** 1 Australian Centre for Health Services Innovation, Centre for Healthcare Transformation, School of Public Health and Social Work, Faculty of Health, Queensland University of Technology, AU

**Keywords:** integrated care, health economics, multi-criteria decision analysis, economic evaluation

## Abstract

This article details a correction to: Blythe R, Carter H, Abell B, Brain D, Dyer C, White N, et al. Application of a Mixed Methods Multi-Criteria Decision Analysis Framework in Integrated Health Care. *International Journal of Integrated Care*. 2022; 22: 19. DOI: http://doi.org/10.5334/ijic.5997.

## Correction

Blythe et al. (2022) [1] was published with an error in the author list. This was due to a communication error during submission by the submitting author.

The original author list was:

Robin Blythe, Hannah Carter, Bridget Abell, David Brain, Carly Dyer, Nicole White, Sanjeewa Kularatna, Steven McPhail

The author list should instead read:

Robin Blythe, Hannah Carter, Bridget Abell, Megan Campbell, David Brain, Carly Dyer, Nicole White, Sanjeewa Kularatna, Steven McPhail

All authors fit the authorship definitions and have confirmed the new list is correct.
